# Prevalence of self-care disability among older adults in China

**DOI:** 10.1186/s12877-022-03412-w

**Published:** 2022-10-01

**Authors:** Yu Guo, Tian Wang, Tingshuai Ge, Quanbao Jiang

**Affiliations:** School of Public Policy and Administration, Institute for Population and Development Studies, Xi’an Jiaotong University, Xi’an, China

**Keywords:** Self-care disability, Older adults, China

## Abstract

**Background:**

Self-care disability among older adults is a global public health issue. However, it lacks the up-to-date information based on nationally representative, more comprehesive data in China.

**Methods:**

Using China’s 2020 population census data, this paper provides a macro-analysis of the prevalence and socio-demographic characteristics of self-care disability among older adults.

**Results:**

25.5 million older adults aged 60 and over participated in the health status survey, of which 48.2% were male, and 51.8% were female. We find that the prevalence of self-care disability among older adults aged 60 and above in China is 2.34%, and the older the population, the higher the prevalence. A higher prevalence was reported by female older adults, rural older adults, and older adults in western China. Single (never married) and widowed older adults are at higher risk of self-care disability. Compared to 2010, the prevalence of self-care disability among older adults decreased. However, the urban-rural difference still exists. Self-care disabled older adults rely mainly on family members for livelihood and mainly cohabitate with them. While pension is an essential source of livelihood for urban older adults with self-care disability, fewer rural self-care disabled older adults rely on pension.

**Conclusion:**

The prevalence of self-care disability among older adults aged 60 and over in China is low and has decreased compared to 2010. Older adults with self-care disability are not a homogeneous group, and they have apparent socio-demographic disparities and regional differences. The Chinese government should continue to reduce inequalities between urban and rural areas, especially in pension and long-term care systems.

## Background

 The rising number of older adults with self-care disabilities is one of the main challenges of population aging. It is estimated that more than 142 million older adults, or 14% of the global population aged 60 and over, are currently unable to meet their basic daily needs [1]. A high number of older people with self-care disabilities puts pressure on healthcare and related services and adds to policy challenges. Therefore, knowing the prevalence and correlates of self-care disability among older adults is a critical first step in addressing their needs through public health services.

The proportion of older adults in China has continued to increase. Between 2010 and 2020, the proportion of the population aged over 60 and above increased from 13.3 to 18.7%. Likewise, over the last decade, the number of people unable to care for themselves increased from 33 million to 40 million between 2010 and 2022 [2, 3]. In China, spouses and children are the primary caregivers for older adults. However, due to a decades-long decline in fertility, family sizes have become smaller, and their functions weakened. With the traditional concept of family care becoming unsustainable, changes become inevitable. The Chinese idiom “*Wei Fu Xian Lao*,” which translates as “getting old before getting rich,” perhaps describes the reality of most older adults in China. Coupled with China’s social security system, which is still very much in infancy, there are considerable challenges for the healthcare systems and the long-term care of older adults with self-care disabilities.

The Chinese government has taken many measures to address the challenges posed by an aging population and self-care disability. At the 19th National Congress of the Communist Party of China, an “active response to an aging population” was proposed and became a national strategic goal. A traditional care system that involves collaboration between family and community was implemented based on resolutions contained in the national strategic goal. Many cities in China adopt “9064” or “9073” care patterns, meaning that 90% of the older adults will be cared for at home, six or seven percent in the community, and four or three percent in institutions. Understanding the prevalence and distribution of older adults with self-care disability in China is sacrosanct to formulating future policies and resource allocation.

In the current study, we primarily utilized 2010 and 2020 Chinese census data to (1) describe the overall prevalence and trend of disability in self-care among older adults. The prevalence is also described relative to socio-demographic characteristics, including age, gender, residence, regions, and marital status. (2) investigate the pattern of care among self-care disabled older adults by analyzing their main sources of livelihood and living arrangements. The analysis findings will equip policymakers, analysts, and researchers to understand the severity of older adults’ self-care disability across demographic characteristics. Similarly, it will assist in designing group-specific policies to address the phenomenon and improves health-related service systems.

Next, we introduce the Chinese context, followed by a description of data and methods. The latter parts include the presentation of results, discussion, and conclusions.

### The Chinese context

China is witnessing a rapid population aging, mainly attributable to two factors. First is the increase in life expectancy due to improvements in science and medicine. Second is the decline in fertility rates due to changes in population trends and, more particularly, China’s series of birth policies. According to China’s 2020 census, the number of people aged 60 and above has increased to 264 million. It is expected to increase to 418 million by 2035, accounting for 39% of the global population of older adults [4]. China has adjusted its one-child policy to curtail the fertility decline, slow down the aging process, and improve the solvency of its pension fund. Now, married couples are allowed to have up to three children. However, it will do little to boost China’s low fertility rate or slow the country’s aging process.

In China, the number of older adults with self-care disabilities is steadily increasing. With the decline of physical and cognitive functioning, an increasing number of older adults have limited activities of daily living [5]. The number of older adults with partial or complete disability increased from 33.0 million to 40.6 million between 2010 and 2015 (the disability rate changed from 19.0–18.3%) [6, 7]. It is estimated that there will be 52 million disabled older adults by 2050, 33.5 million in urban areas, and 18.5 million in rural areas, respectively [8].

There is a huge disparity between the demand and supply of healthcare services for older adults in China. It is estimated that the life expectancy of people aged 65 is 16.3 years, including 8.7 years of healthiness, 4.8 years with difficulty in IADL, and 2.8 years with difficulty in ADL [9]. Traditionally, spouses and children are the main caregivers for older adults. However, with the increase in life expectancy, it is estimated that 41 million older adults were widowed in China in 2010 [9]. With the number of children and family size shrinking, the traditional family function of old age support has weakened. Combined with the change from the traditional ideology of family support, an increasing number of people believe that the government and society should bear the responsibility of caring for older adults [10]. Caring for older adults has become a prominent and complex issue in China [9].

On the one hand, older adults in China “get old before getting rich,” whereas the pension system is overly dependent on government finance, which bears at least 80% of the payment [11]. The fund’s sustainability will inevitably face challenges with China’s rapid aging. It is estimated that the cost of long-term care for older adults in rural areas may exceed 2 trillion dollars in 2050 [12]. On the other hand, medical insurance and nursing systems cannot cover the majority of the older adults who are disabled from self-care [13]. Older adults with low income or without income at all, especially those in rural areas, have to rely on family support in the future and cannot afford to pay for their care.

## Methods

### Data

This paper analyzed data extracted from China’s 2020 Population Census. The post-enumeration survey indicated an under-reporting rate of 0.05%. The data provided fresh insights into China’s recent demographic trends and revealed vital information that would contribute to the country’s policymaking over the next 10 years. A 10% sample of randomly selected households completed the long-form questionnaire. 25.5 million older adults aged 60 and over participated in the health status survey, of which 48.2% were male, and 51.8% were female. We also used China’s 2010 population census data to compare and assess trends.

### Measurement

The long-form questionnaire surveyed information on self-reported health status, socio-demographic characteristics (age, gender, residence, regions, and marital status), the main source of livehood, and living arrangement.

The self-care ability of the older adult population aged 60 and above was measured using self-reported health status, classified as Good, Fair, Poor—with self-care ability, and Poor—without self-care ability. “Poor—without self-care ability” means the health status has been poor in the past month and unable to take care of daily life, such as eating, dressing, and moving around. The group without self-care ability was regarded as self-care disabled and taken as the focus of this study.

Marital status is classified as single, widowed, divorced, and married with the spouse. The main sources of livelihood were categorized as family members, retirement pension, unemployment insurance, minimum living security, labor income, property income, and other income. Since only a few older people rely on unemployment insurance and subsistence security, and both come mainly from government benefits, we have combined them as government subsistence allowance. Also, property income was combined with the “other income” category for the convenience of analysis. Living arrangements include living with spouse and children, living with spouse only, living with children only, living alone, and living in elderly care institutions.

### Statistical analysis

 The prevalence of self-care disability is defined as the proportion of older adults unable to care for themselves. To calculate the self-care disability prevalence in this study, we divide the number of older adults with self-care disability by the total population of older adults aged 60 and above. The overall prevalence and the prevalence by age, sex, residence, region, and marital status were reported. To examine the pattern of care among self-care disabled older adults in China, we also reported proportions relative to each form of the sources of livelihood and living arrangement.

## Results

### General trends

In 2020, the prevalence of self-care disability among older adults aged 60 years and above was 2.34%, lower than 2.95% in 2010.

**Fig. 1:**
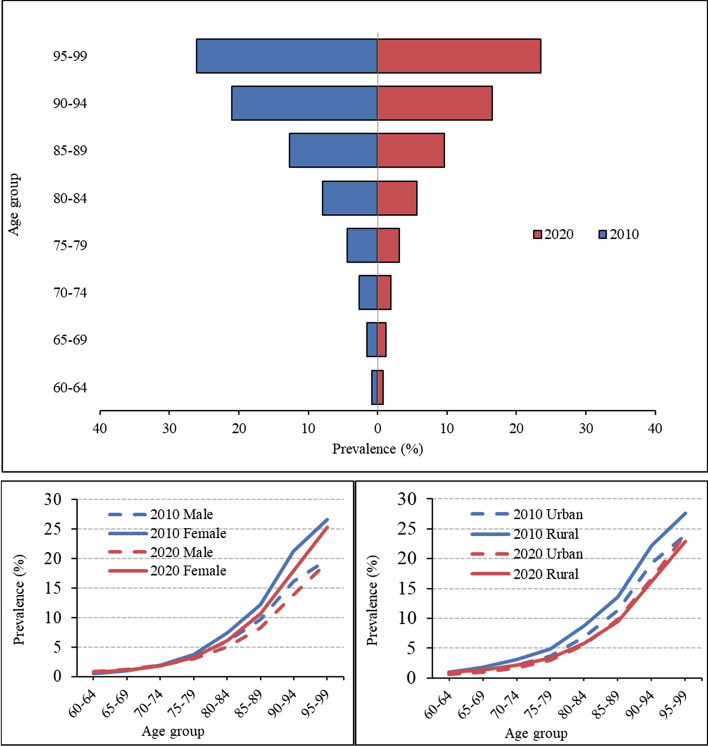
General trends of prevalence in 2010 and 2020. **a** Prevalence by age group; **b** Prevalence by age group and gender; **c** Prevalence by age group and residence

Figure 1A shows that the prevalence of self-care disability increases with age. The prevalence among older adults aged 80 and above is higher than 5%, and among those over 90 years, it is higher than 15%. The reported prevalence decreased in 2020 compared with that in 2010.

Generally, the prevalence of self-care disability is higher among women than among men, as indicated in Fig. 1B. For older adults aged below 75 years, the prevalence is similar between men and women. While among those aged 75 years and above, a higher prevalence is reported among women than among men, and this difference gradually increases with age. The reported prevalence decreased for both genders in 2020 compared with 2010.

### Residence difference

Figure 1C presents differences based on residence. A higher prevalence of self-care disability was generally reported among older adults in rural areas than in urban areas. However, for the oldest-old aged 85 and above, the prevalence in rural areas is higher than in urban areas. The urban-rural disparity has shrunk significantly compared with that in 2010.

### Regional difference

**Fig. 2:**
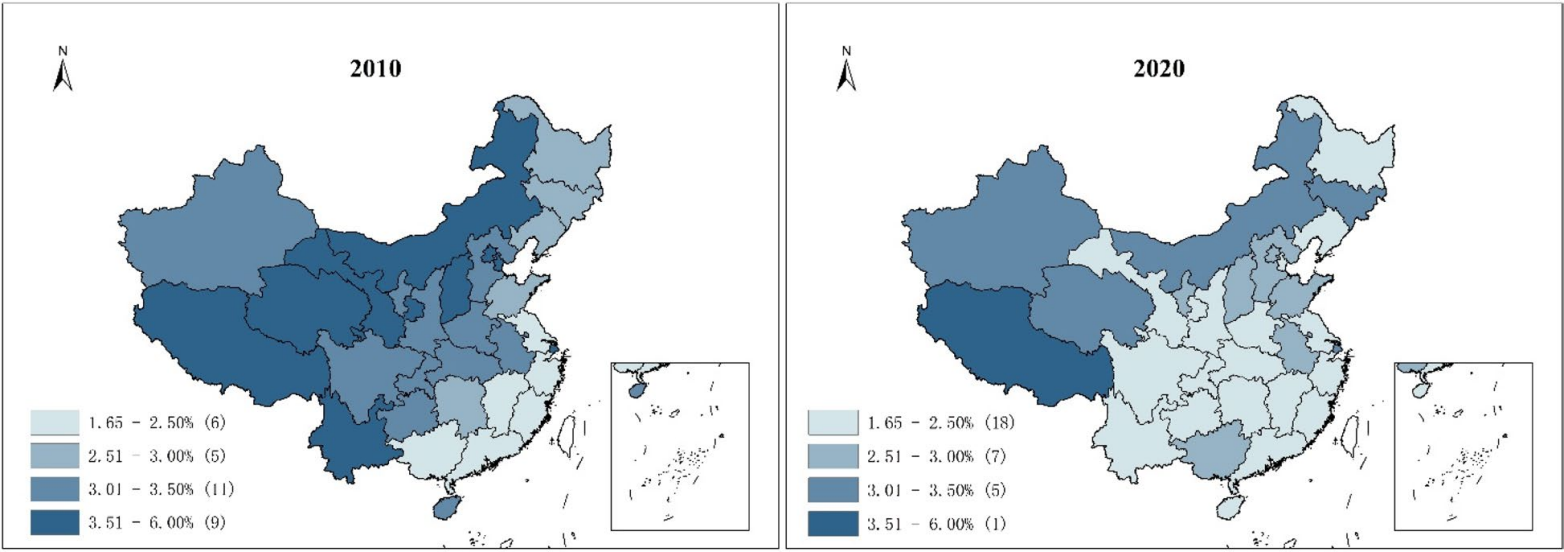
Prevalence by province. **a** Prevalence by province in 2010; **b** Prevalence by province in 2020

Figure 2 shows the regional difference in the prevalence of self-care disability among older adults. Self-care disability is more prevalent in Inner Mongolia and western regions than in the southeast coastal region, which is more developed in China. In 2020, there are 18 provinces where the prevalence is lower than 2.5%. The number of provinces with more than 3% prevalence decreased from 20 (in 2010) to 6, including Inner Mongolia, Shanghai, Qinghai, Jilin, Xinjiang, and Tibet. The prevalence decreased significantly in the central, western (except Xinjiang and Tibet) and southwestern regions compared with 2010.

### Marital status

**Fig. 3:**
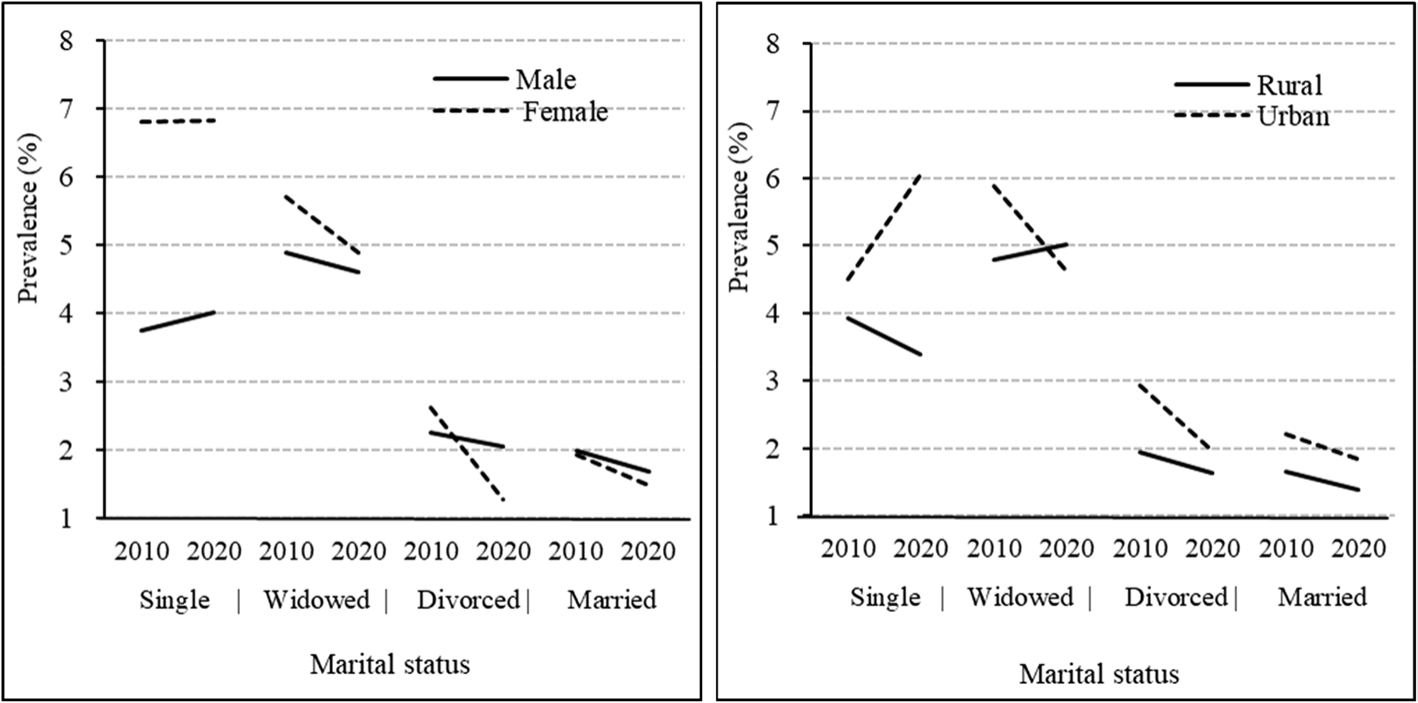
Prevalence by marital status. **a** Prevalence by marital status and gender; **b** Prevalence by marital status and residence

Figure 3 provides the prevalence of self-care disability among older adults by marital status. Among all marital statuses, never married and widowed older adults reported the highest prevalence of self-care disability compared with other groups. The prevalence of self-care disability among never-married women older adults is approximately twice that of men. Compared with 2010, the prevalence of self-care disability increased among urban never-married older adults and rural widowed older adults.

### Main source of livelihood

**Fig. 4:**
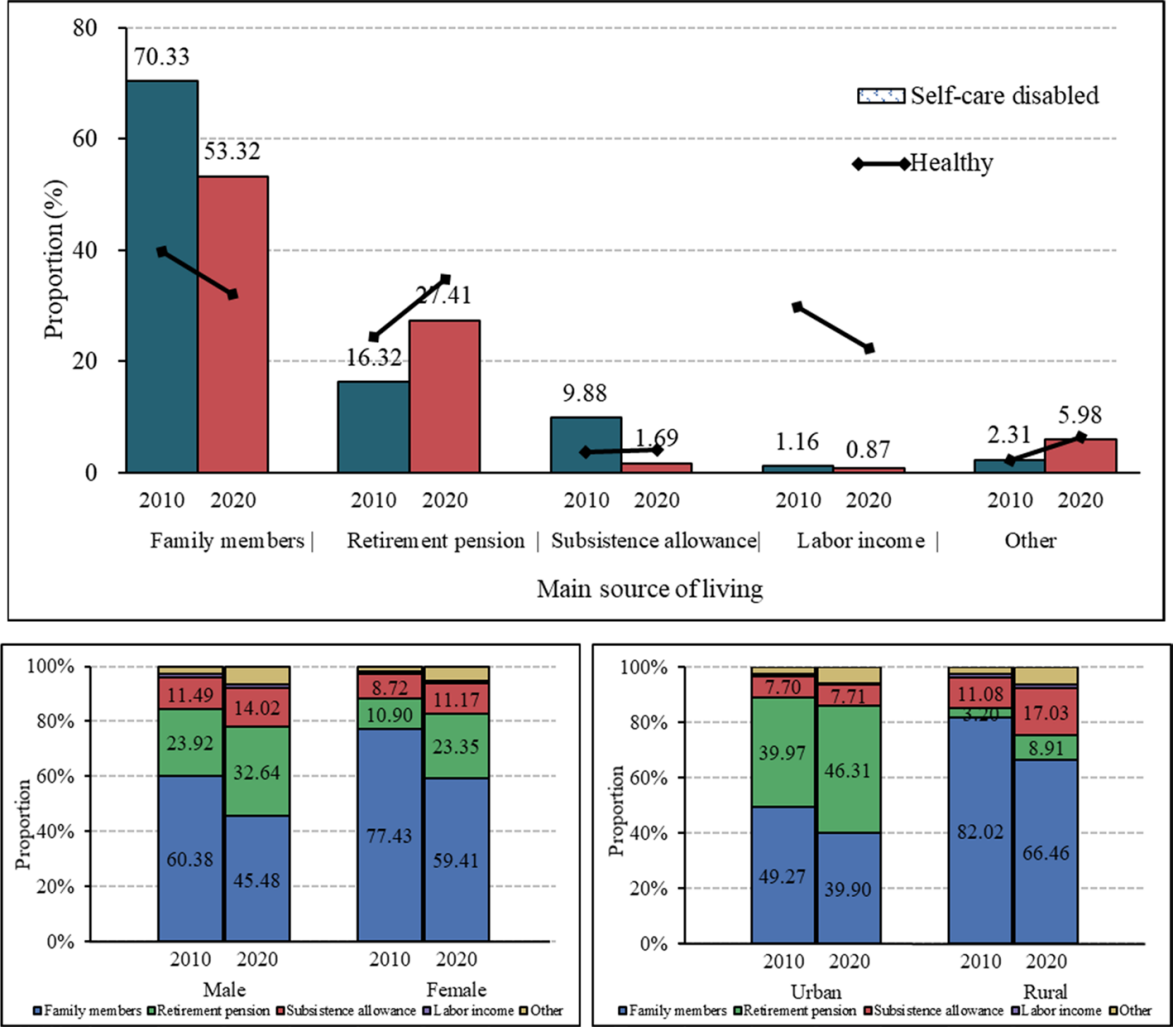
Main source of livelihood. **a** Main source of livelihood for self-care disabled and healthy older adults; **b** Main source of livelihood by gender; **c** Main source of livelihood by residence

Figure 4A shows the proportion of older adults by the main sources of livelihood, where the bar chart and line chart denote the proportion of self-care disabled and healthy older adults in 2010 and 2020, respectively. Self-care disabled older adults mainly depend on family members, while healthy older people largely rely on their labor income. Compared with 2010, for both self-care disabled and abled older adults, the proportion of people relying on retirement funds has increased. Pensions have exceeded labor income and become the main source of livelihood for self-care abled older people. Among older adults who reported self-care disability, women mainly depended on family support, while men could also rely on pensions (Fig. 4B).

 In urban areas, pensions and other family members are the main sources of livelihood for older adults with self-care disability. However, in rural areas, family members are the main source of livelihood (Fig. 4C). Compared to 2010, the proportion of people depending on subsistence allowance increased in rural areas.

### Living arrangement

**Fig. 5:**
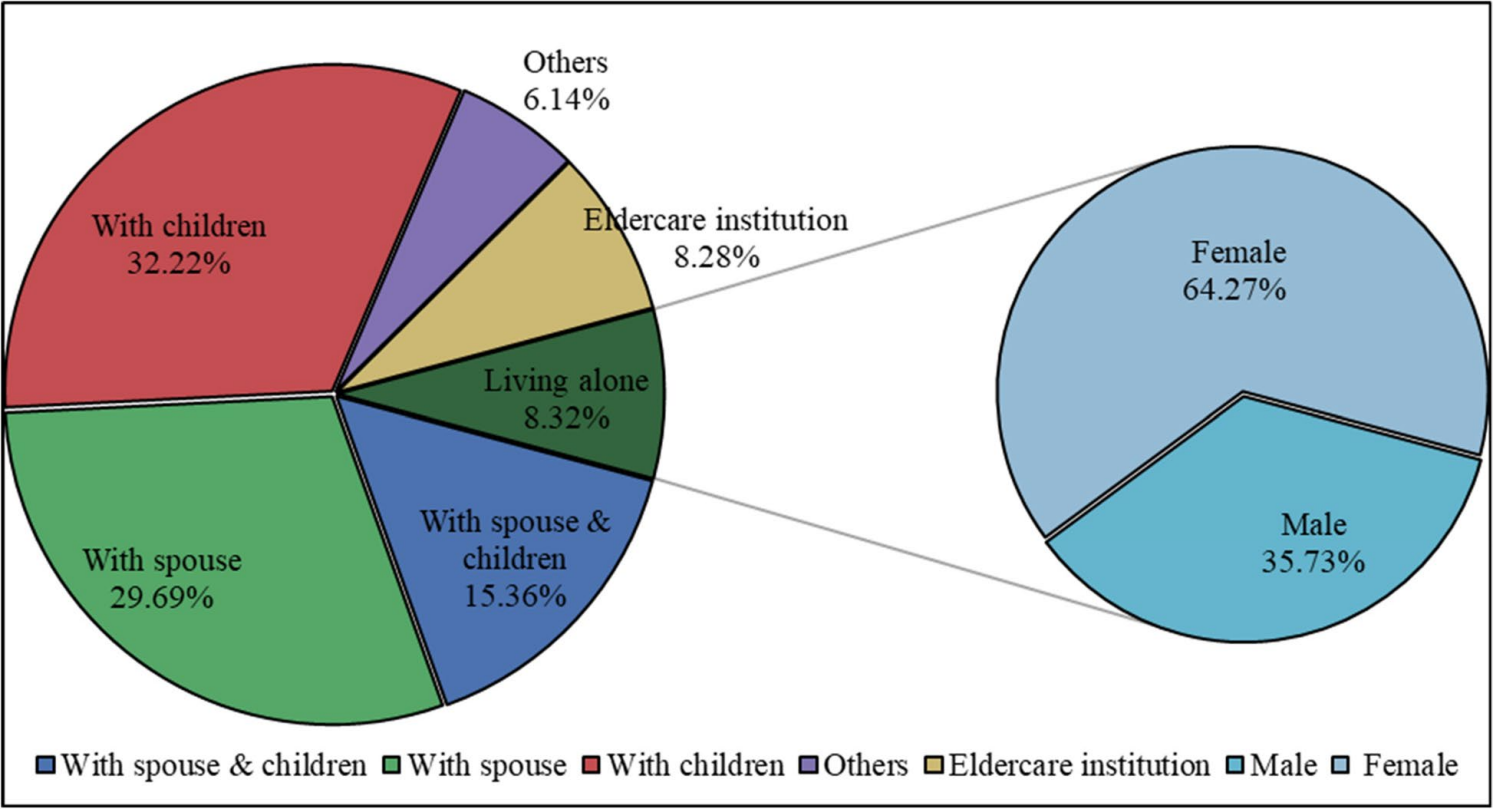
Living arrangements (%)

Figure 5 provides information on self-care disabled older adults based on living arrangements. About 77.27% live with their spouse or children, 8.32% live alone, and are mostly women. Only a small percentage of older adults with self-care disability live in elderly care institutions. A higher proportion of women and older persons in rural areas live with their families. In urban areas, the proportion of older adults with self-care disability living in elderly care institutions is higher than that in rural areas (Table 1).

**Table 1:** Living arrangements by gender and residence (%)

Living arrangement	Gender		Residence	
	Male	Female	Urban	Rural
With spouse and Children	20.75	11.17	15.53	15.19
With spouse	38.12	23.14	26.52	32.79
With children	19.10	42.41	30.87	33.54
Living alone	6.79	9.50	8.08	8.54
Eldercare institution	8.53	8.08	12.86	3.79
Others	6.70	5.70	6.14	6.13
Total	100.00	100.00	100.00	100.00

## Discussion

Self-care disability among older adults is a global public concern, especially in developing countries intertwined with rapid aging and inadequate long-term care. With the latest available data, we analyzed the prevalence and socio-demographic disparities in the prevalence of self-care disability among Chinese older adults. The findings are as follows.

We found that the prevalence of self-care disability among older adults aged 60 and above was 2.34% in 2020, a slight decrease from 2010. However, due to China’s huge older adults demography and its rapidly aging process, the absolute number of self-care disabled older adults is still growing.

The risk of self-care disability increases with age, especially after the age of 80. This could result from the degeneration of body organs and the onset of certain chronic diseases as people age. However, as sanitation and medical conditions improve and life expectancy increases in China, older persons can live longer despite their diminished ability to care for themselves.

Between the ages of 60 and 75 years, both men and women reported an equal prevalence of self-care disability. After 75 years of age, women are less able to self-care than men, with the gap widening with age. Women’s life expectancy at birth is 80.5, while men’s is 74.7 in China [14]. China has a higher proportion of older women, and women are more likely to survive regardless of their health or ability to care for themselves [15].

People living in rural areas reported a higher prevalence of self-care disability than in urban areas, but the difference is gradually narrowing. With China’s urbanization, more medical resources have been invested in urban areas, which leads to the urban-rural inequality in the health status of older adults [16]. Besides, nursing and long-term care resources are also more abundant in urban areas, so older adults with self-care ability in urban areas are more likely to enjoy convenient medical services and survive longer. In the past decade, the prevalence of self-care disability among older adults aged 85 and above has exceeded that in rural areas.

The prevalence of self-care disability is higher among older adults in the western and central regions. China implemented the economic reform and opening-up policy at the end of the 1970s, which brought enormous economic growth to the southeastern coastal regions, attracting many employment-related migrations [16]. Central and western regions in China have fallen behind the eastern region not only in socioeconomic development and population aging but also in medical resources and long-term care resources. All these factors are intertwined, leading to regional disparities in the prevalence of self-care disabilities.

The prevalence of self-care disability is highest among never married and widowed older adults in China. Spouses and family members play a unique role in Chinese people’s health [17]. Traditionally, China is a country with universal marriage, but this characteristic is becoming subject to changes due to socioeconomic development and ideological change [18, 19]. Being single for life has a significant impact on the health of Chinese people. This study found that single people also had a higher risk of self-care disability as they entered old age. China has the second-largest number of widowed older adults globally [20]. The health of older adults will gradually deteriorate after being widowed, and the risk of stroke or dementia will gradually increase, which are the main reasons for self-care disability [21–23].

The proportion of older adults whose main source of livelihood comes from family members has decreased, while those relying on the pension have substantially increased. China adopts a family-based care pattern, and most older adults with self-care disability live with their spouses or children, which is particularly common in rural areas. However, with the shrinking family size and the rapid aging process, family support and older adult care resources gradually decrease. An increasing number of people believe that the government should shoulder the responsibility of supporting older adults [10].

 In addition to family members, pensions are another primary income source for older adults with self-care disability in urban areas. However, most older adults can only live on family members in rural areas. Although the pension system in China has been reformed to adjust for urban-rural income inequality in 2015, gaps remain in pension participation and access between rural and urban areas [24].

Our findings also have important policy implications. First, given that most disabled older adults still live with their families, we recommend that government establish guidelines and prioritize resources for family-based care. Currently, most policies focus on developing institutional care, resulting in many unoccupied facilities. A combination of training, incentives, and respite service policies is needed to help informal family caregivers become more efficient and to reduce the burden on caregivers. Second, the long-term care funding mechanism should be more equitable and targeted. A step was taken in this regard with the 2016 launch of long-term care insurance (LTCI), a pilot program intended to support Chinese older people with affordable care services in specific cities. Based on this study, we recommend that more rural areas and western regions be included in the pilot program. Equally important is that women, single and widowed older adults should be given priority inclusion in the LCTI to ensure their guaranteed access to the services. The third is to adopt preventive measures to delay self-care disability. The Chinese government has already initiated “*Yi Yang Jie He*” to integrate health and long-term care services for the generality of older adults. Our study suggests this effort should be more specifically targeted at men and older adults living in rural areas. Fourth, the current study provides some results data on prevalence and correlates based on the 2020 census, which may be valuable in strengthening future research. Based on the data, further research could employ correlation and casualization analysis with available survey data, to estimate and project the need for long-term care beds, staff, and budget. At the same time, given the long-standing divide between urban and rural areas, continuous research is also required to monitor the gap and inequalities in resources and needs.

## Conclusion

The prevalence of self-care disability among older adults aged 60 and over in China is low and has decreased compared to 2010. Older adults with self-care disability are not a homogeneous group, and they have apparent socio-demographic disparities and regional differences. In China, self-care disabled older adults mainly cohabitate and live on their families. It needs to be noted that urban-rural inequality still exists in retirement pensions. Although a large proportion of older adults in urban areas can rely on pensions, only a small percentage in rural areas enjoy retirement benefits. In the future, the Chinese government should continue to improve social security and health-related services targeted at older people with self-care disability, while reducing inequalities between urban and rural areas, especially in pension and long-term care systems.

## Data Availability

The datasets used and/or analysed during the current study are available from the corresponding author on reasonable request.
